# The impact of perioperative risk factors on long-term survival after radical cystectomy: a prospective, high-volume cohort study

**DOI:** 10.1007/s00345-024-04887-5

**Published:** 2024-03-15

**Authors:** Nikolaos Pyrgidis, Gerald B. Schulz, Yannic Volz, Benedikt Ebner, Severin Rodler, Thilo Westhofen, Lennert Eismann, Julian Marcon, Christian G. Stief, Friedrich Jokisch

**Affiliations:** Department of Urology, University Hospital, LMU Munich, Marchioninistraße 15, 81377 Munich, Germany

**Keywords:** Bladder cancer, Cystectomy, Survival, Perioperative outcomes

## Abstract

**Introduction:**

Radical cystectomy (RC) is the gold standard for muscle-invasive bladder cancer. Nevertheless, RC is associated with substantial perioperative morbidity and mortality. We aimed to evaluate the role of important perioperative risk factors in predicting long-term survival after RC.

**Methods:**

An analysis of the prospective cohort of patients undergoing open RC from 2004 to 2023 at our center was performed. Patients who died within one month after RC were excluded from the study. A univariate and multivariable Cox regression analysis was performed to assess the role of sex, age, urinary diversion, preoperative values of creatinine and hemoglobin, first-day postoperative values of CRP, leucocytes, and thrombocytes, perioperative Clavien-Dindo complications, perioperative chemotherapy, admission to the intensive or intermediate care unit, as well as type of histology, pathologic T-stage, positive lymph nodes, and positive surgical margins on predicting the long-term overall survival after RC. For all analyses hazard ratios (HRs) with the corresponding 95% confidence intervals (CIs) were estimated.

**Results:**

A total of 1,750 patients with a median age of 70 years (IQR: 62–76) were included. Of them, 1,069 (61%) received ileal conduit and 650 (37%) neobladder. Overall, 1,016 (58%) perioperative complications occurred. At a median follow-up of 31 months (IQR: 12–71), 884 (51%) deaths were recorded. In the multivariable Cox regression analysis, increasing age (HR: 1.03, 95%CI: 1.02–1.04, p < 0.001), higher preoperative creatinine values (HR: 1.27, 95%CI: 1.12–1.44, p < 0.001), lower preoperative hemoglobin values (HR: 0.93, 95%CI: 0.89–0.97, p = 0.002), higher postoperative thrombocyte values (HR: 1.01, 95%CI: 1.01–1.02, p = 0.02), Clavien-Dindo 1–2 complications (HR: 1.26, 95%CI: 1.03–1.53, p = 0.02), Clavien-Dindo 3–4 complications (HR: 1.55, 95%CI: 1.22–1.96, p < 0.001), locally advanced bladder cancer (HR: 1.29, 95%CI: 1.06–1.55, p = 0.009), positive lymph nodes (HR: 1.74, 95%CI: 1.45–2.11, p < 0.001), and positive surgical margins (HR: 1.61, 95%CI: 1.29–2.01, p < 0.001) negatively affected long-term survival.

**Conclusion:**

Beside increased age and worse oncological status, impaired renal function, lower preoperative hemoglobin values, higher postoperative thrombocyte values, and perioperative complications are independent risk factors for mortality in the long term in patients undergoing open RC.

## Introduction

Radical cystectomy (RC) remains the gold-standard for the treatment of patients with muscle-invasive or high-risk non-muscle-invasive bladder cancer [1]. Despite advancements in surgical techniques and improvements in inpatient care, RC continues to be associated with high rates of perioperative morbidity and mortality [2, 3]. Current evidence suggests that more than two-thirds of all patients undergoing RC experience perioperative complications [4]. Overall, gastrointestinal (19%), infectious (17%), cardiovascular (9%), respiratory (7%), and genital or urologic (7%) complications are the most common [5]. Of them, approximately 20% may be life-threatening, leading to surgical reinterventions or unplanned admissions to the intensive care unit and potentially resulting in perioperative mortality rates of about 8% after open RC [6, 7].

It has been postulated that, in patients undergoing major oncological operations, the occurrence of perioperative complications may not only negatively affect perioperative mortality but it is also a risk factor for worse long-term survival, even in patients who survived the postoperative period [8]. Indeed, recent data from other major oncological operations suggest an adverse impact of perioperative complications on long-term survival outcomes [9, 10]. It has been hypothesized that perioperative complications may lead to immunosuppression and further immunomodulatory effects, which can pave the way for early cancer recurrence and, in turn, negatively affect long-term survival [11]. Based on the previous notion, it seems that perioperative blood transfusion can cause transient immunosuppression which may induce hematogenic tumor cell circulation in cancer patients, facilitating distal seeding of circulating tumor cells [12]. Furthermore, patients with severe perioperative complications may be unable to receive adjuvant systemic therapy, which may also negatively affect their long-term survival [13].

Even though it is of utmost importance to identify the perioperative risk factors that may lead to worse prognosis in the long-term in patients undergoing RC, studies on the matter are lacking. Within this framework, we aimed to evaluate the role of major perioperative risk factors in predicting long-term survival after open RC.

## Materials and methods

### Study design

Since 2004 data from a prospective cohort of all patients undergoing open RC at our University Urology Department were collected. This study was approved by the corresponding institutional review (Reference number: 20–179) and is undertaken according to the ethical standards of the Declaration of Helsinki. Written informed consent is obtained from all patients and these findings are reported based on the STROBE statement for cohort studies [14]. We included all patients undergoing open RC between 2004 and 2023, on whom the perioperative complications were reported. We excluded patients who underwent RC for non-oncological reasons, as well as those who died within one month after RC.

### Primary outcome and statistical analysis

The primary outcome of the present analysis was to determine the role of major perioperative factors in predicting long-term survival after RC. For this purpose, a univariate Cox regression analysis was performed to assess the effect of sex, age, urinary diversion, preoperative values of creatinine and hemoglobin, first-day postoperative values of CRP, leucocytes, and thrombocytes, perioperative Clavien-Dindo complications [15], perioperative chemotherapy, admission to the intensive or intermediate care unit, as well as type of histology, pathologic T-stage, positive lymph nodes, and positive surgical margins on predicting the long-term overall survival after RC. The included independent major perioperative factors were chosen based on importance and clinical relevance after consensus among the study investigators. These perioperative factors were added to a multivariable Cox regression analysis to evaluate their effect on long-term survival after open RC. The proportional hazards assumption was evaluated both statistically with the goodness of fit test and graphically with Kaplan–Meier curves. We resolved any discrepancies between the two tests through the construction of observed versus predicted curves and log-minus-log plots.

Continuous variables were summarized as median with interquartile range (IQR) and categorical variables as frequencies with proportions. The corresponding comparisons were performed with the Mann–Whitney test and the chi-squared test. The Kaplan–Meier curves with the log-rank test were also used to evaluate the effect of impaired renal function on overall survival. For all survival outcomes, we estimated hazard ratios (HRs) with the corresponding 95% confidence intervals (CIs). A two-sided p-value < 0.05 was considered statistically significant for all estimates. All analyses were undertaken with the R statistical software (version 3.6.3).

## Results

### Baseline characteristics

A total of 1,883 patients underwent open RC at our institution for oncological reasons. Of them, 123 patients (6.5%) patients died within one month after RC. Thus, 1,750 patients were included in the present analysis. Their median age was 70 years (IQR: 62–76), their median BMI was 26 kg/m^2^ (IQR: 23–29) and 1,283 (73%) patients were male. A total of 826 (55%) patients were smokers, 1,073 (62%) had hypertension, and 678 (39%) had diabetes. Of them, 1,069 (61%) received an ileal conduit and 650 (37%) an orthotopic ileal neobladder. The operative time of RC was 229 min (IQR: 186–279), the intraoperative blood loss 500 ml (IQR: 300–963), and the median length of hospital stay was 20 days (IQR: 16–24). Of note, 134 (7.7%) patients presented variant histology, 947 (63%) locally advanced bladder cancer (≥ pT3), 358 (27%) positive lymph nodes (pN +) and 224 (15%) positive surgical margins at the time of open RC. The median preoperative and first-day postoperative creatinine values were 1.1 mg/dl (IQR: 0.9–1.3). Despite worse oncological findings in the present cohort, only 515 (29%) patients underwent perioperative (neoadjuvant or adjuvant) chemotherapy within three months from the operation.

Overall, 1,016 (58%) patients experienced at least one perioperative complication during hospital stay. Based on the Clavien-Dindo classification, most complications were grade 1 or 2 (609, 66%). Moreover, 534 (31%) patients required transfusion with a median number of 2 blood units (IQR: 1–3), and 915 (53%) were admitted postoperatively to the intensive or intermediate care unit for a median duration of 2 days (IQR: 2–3). Other common complications included urinary and surgical wound infections, ileus, and cardiopulmonary complications. As expected, patients experiencing perioperative complications were older (p < 0.001), underwent urinary diversion with orthotopic ileal neobladder less frequently (p < 0.001), had worse ASA score (p < 0.001), had more often diabetes (p < 0.001), hypertension (p < 0.001) and heart disease (p < 0.001), whereas their operation lasted longer (p < 0.001) with more blood loss (p < 0.001) and worse histological findings (p < 0.001). Accordingly, patients with perioperative complications presented a statistically significantly worse preoperative and first-day creatinine, CRP, hemoglobin, leucocyte, and thrombocyte values. The baseline characteristics of the whole study cohort based on the occurrence of at least one perioperative complication during hospital stay are available in Table 1.

**Table 1:** Baseline characteristics of all patients undergoing radical cystectomy

Characteristic	Overall, n = 1,750
Age (years)	70 (62–76)
Males	1,283 (73%)
BMI	26 (23–29)
Smokers	826 (55%)
Alcohol consumption	570 (40%)
Heart disease	590 (34%)
Hypertension	1,073 (62%)
Diabetes	678 (39%)
ASA	
1	37 (2%)
2	537 (31%)
3	1,121 (65%)
4	28 (2%)
Urinary diversion	
Ileal Conduit	1,069 (61%)
Neobladder	650 (37%)
Pouch	16 (0.9%)
Ureterocutaneostomy	15 (0.9%)
Operative time (minutes)	229 (186–279)
Blood loss (ml)	500 (300–963)
Histology	
Urothelial cancer	1,602 (92%)
Variant histology	134 (7.7%)
T after cystectomy	
≤ T2	562 (37%)
≥ T3	947 (63%)
Positive lymph nodes	358 (27%)
Positive surgical margins	224 (15%)
Hospital stay (days)	20 (16–24)
Perioperative chemotherapy	515 (29%)
Preoperative creatinine (mg/dl)	1.1 (0.9–1.3)
Preoperative CRP	0.5 (0.2–1.4)
Preoperative leucocytes	8 (6–9)
Preoperative hemoglobin	13 (12–15)
Preoperative thrombocytes	258 (209–319)
1-day postoperative creatinine (mg/dl)	1.1 (0.9–1.3)
1-day postoperative CRP	9 (6–12)
1-day postoperative leucocytes	10 (8–12)
1-day postoperative hemoglobin	10 (9–11)
1-day postoperative thrombocytes	180 (144–226)

Values are presented as median (interquartile range) or n (%)

*ASA* American Society of Anesthesiology, *BMI* Body Mass Index

### Perioperative risk factors and long-term survival

At a median follow-up of 31 months (IQR: 12–71), 884 (51%) deaths occurred. Of them, 598 (59%) were reported in the group with perioperative complications and 286 (39%) in the group with no perioperative complications (p < 0.001). In the univariate Cox regression analysis, all assessed major perioperative risk factors (except for first-day postoperative leucocyte values) were associated with statistically significant worse long-term survival. Subsequently, the effect of these risk factors on survival was assessed through a multivariable Cox regression analysis.

In the multivariable Cox regression analysis, increased age (HR: 1.03, 95% CI: 1.02 to 1.04, p < 0.001), higher preoperative creatinine values (HR: 1.27, 95% CI: 1.12 to 1.44, p < 0.001), lower preoperative hemoglobin values (HR: 0.93, 95% CI: 0.89 to 0.97, p = 0.002), higher postoperative thrombocyte values (HR: 1.01, 95% CI: 1.01 to 1.02, p = 0.02), Clavien-Dindo 1–2 complications (HR: 1.26, 95% CI: 1.03 to 1.53, p = 0.024), Clavien-Dindo 3–4 complications (HR: 1.55, 95% CI: 1.22 to 1.96, p < 0.001), locally advanced bladder cancer (HR: 1.29, 95% CI: 1.06 to 1.55, p = 0.009), positive lymph nodes (HR: 1.74, 95% CI: 1.45 to 2.11, p < 0.001), perioperative chemotherapy (HR: 1.32, 95% CI: 1.09 to 1.59, p = 0.004) and positive surgical margins (HR: 1.61, 95% CI: 1.29 to 2.01, p < 0.001) displayed a negative effect on long-term survival. On the contrary, patients undergoing neobladder presented better long-term survival compared to ileal conduit (HR: 0.78, 95% CI: 0.64 to 0.95, p = 0.014). The univariate and multivariable Cox regression analysis is presented in Table 2. In patients who required chemotherapy, the administration of chemotherapy was associated with better overall survival in the multivariate analysis (HR: 0.86, 95% CI: 0.71 to 0.95, p = 0.008) compared to omission of chemotherapy.

**Table 2:** Univariate and multivariate Cox regression models for the effect of major perioperative factors on survival in patients undergoing radical cystectomy

Characteristic	Univariate			Multivariable		
	HR	95% CI	p-value	HR	95% CI	p-value
Males	0.77	0.66, 0.89	** < 0.001**	1.03	0.85, 1.25	0.8
Age	1.03	1.02, 1.03	** < 0.001**	1.03	1.02, 1.04	** < 0.001**
Preoperative creatinine	1.47	1.36, 1.58	** < 0.001**	1.27	1.12, 1.44	** < 0.001**
Preoperative hemoglobin	0.84	0.81, 0.86	** < 0.001**	0.93	0.89, 0.97	**0.002**
1-day postoperative CRP	1.03	1.01, 1.04	** < 0.001**	1.01	0.99, 1.02	0.5
1-day postoperative leucocytes	1.01	0.99, 1.03	0.2	0.98	0.96, 1.01	0.2
1-day postoperative thrombocytes	1.02	1.01, 1.03	**0.004**	1.01	1.01, 1.02	**0.02**
Clavien-Dindo complications						
No	–	–		–	–	
Grade 1–2	1.76	1.51, 2.05	** < 0.001**	1.26	1.03, 1.53	**0.024**
Grade 3–4	2.13	1.78, 2.55	** < 0.001**	1.55	1.22, 1.96	** < 0.001**
Admission to the intermediate or intensive care unit	1.86	1.62, 2.14	** < 0.001**	1.19	0.99, 1.43	0.06
T after cystectomy						
≤ T2	–	–		–	–	
≥ T3	1.94	1.66, 2.27	** < 0.001**	1.29	1.06, 1.55	**0.009**
Positive lymph nodes	2.6	2.21, 3.04	** < 0.001**	1.74	1.45, 2.11	** < 0.001**
Perioperative chemotherapy	1.73	1.5, 1.99	** < 0.001**	1.32	1.09, 1.59	**0.004**
Positive surgical margins	2.97	2.49, 3.53	** < 0.001**	1.61	1.29, 2.01	** < 0.001**
Urinary diversion						
Ileal Conduit	–	–		–	–	
Neobladder	0.44	0.38, 0.51	** < 0.001**	0.78	0.64, 0.95	**0.014**
Pouch	0.39	0.16, 0.95	**0.038**	0.38	0.09, 1.53	0.2
Ureterocutaneostomy	0.55	0.23, 1.34	0.2	0.62	0.15, 2.57	0.5
Histology						
Urothelial cancer	–	–		–	–	
Variant histology	1.29	1.01, 1.64	**0.04**	1.03	0.77, 1.39	0.8

All variables assessed in the univariate Cox regression analysis were also assessed in the multivariate Cox regression analysis

*CI* confidence interval, *HR* hazard ratio

**Fig. 1:**
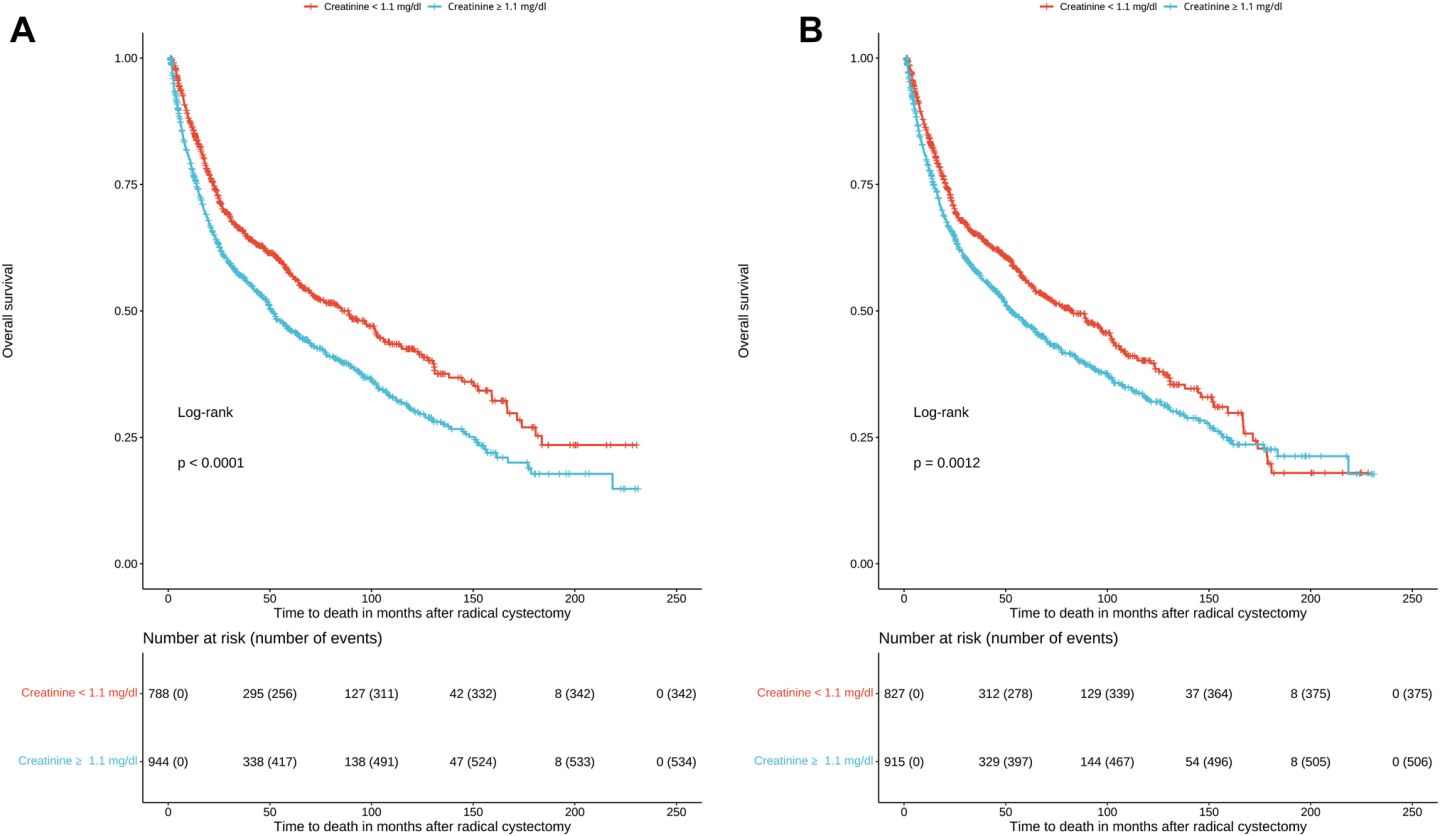
**A** Kaplan-Maier curve for overall survival in patients undergoing radical cystectomy based on preoperative renal function. **B** Kaplan-Maier curve for overall survival in patients undergoing radical cystectomy based on first-day postoperative renal function

Given that the median preoperative and first-day creatinine values of the study cohort were 1.1 mg/dl, we evaluated whether patients with increased creatinine values before RC and on the first day after RC presented worse long-term overall survival. Based on the log-rank test and the corresponding Kaplan–Meier curves, impaired preoperative and fist-day postoperative renal function was independently associated with worse overall survival (p < 0.01). The corresponding Kaplan–Meier curves are depicted in Fig. 1.

## Discussion

The findings of the present high-volume single-center cohort study suggest that major preoperative risk factors and perioperative complications are associated with worse long-term survival in patients undergoing open RC. More specifically, after adjusting for major further parameters, increasing age was associated with 3% poorer long-term survival, preoperative anemia with 8%, higher postoperative thrombocyte values with 1%, impaired renal function with 27%, locally advanced bladder cancer with 29%, lymph node invasion with 74%, and positive surgical margins with 61%. Accordingly, the occurrence of mild (grade 1–2) Clavien-Dindo complications was associated with 26% poorer long-term survival and the occurrence of severe (grade 3–4) Clavien-Dindo complications was associated with 55%. Interestingly, it seems that preoperative and first-day creatinine ≥ 1.1 mg/dl is an independent risk factor for worse long-term survival in patients undergoing RC.

It should be highlighted that impaired renal function is also associated with worse survival outcomes in the general population [16]. In patients undergoing RC, a substantial proportion develops renal function deterioration in the long term, irrespective of the type of urinary diversion [17]. Nevertheless, no studies have assessed the impact of impaired preoperative and first-day renal function on long-term survival [18]. Based on our findings, even though creatinine remained stable on the first postoperative day compared to its preoperative values, both creatinine values were associated with poorer survival.

In the multivariable survival analysis, the occurrence of any perioperative complication was independently associated with poorer long-term survival. The latter was even worse in patients experiencing severe perioperative complications. Although studies on the matter are scarce, a recent retrospective multi-institutional study from Japan indicated that patients experiencing severe postoperative complications had shorter overall and recurrence-free survival [19]. It seems that, to date, there is an increasing trend toward the centralization of bladder cancer care and RC, in an attempt to reduce postoperative complications and, in turn, provide better long-term outcomes [20].

Further perioperative parameters such as lower preoperative hemoglobin values and worse histological findings (locally advanced bladder cancer, positive lymph nodes and positive surgical margins) were also associated with worse long-term survival. Preoperative anemia and subsequent need for transfusion are well-established risk factors for both short- and long-term mortality after RC [21, 22]. Accordingly, aggressive bladder cancer also independently affects long-term survival [23, 24]. Interestingly, in the present analysis, variant histology was not associated with worse long-term survival. The latter may simply be the result of residual confounding with other major risk factors. Besides, relevant studies on the matter suggest that variant histology negatively affects both overall and recurrence-free survival [25]. Accordingly, urinary diversion with neobladder was associated with better overall survival compared to ileal conduit. The latter might be probably explained due to the better health and oncological status of patients receiving orthotopic neobladder [26].

It should be stressed that the findings of the present analysis were mitigated by some limitations relevant to its single-center design. We limited our analysis to patients undergoing open RC, and, thus, our findings may not be extrapolated to other departments that perform other surgical approaches. Importantly, even though we assessed the effect of preoperative and first-day creatinine values on long-term survival, we could not provide data on the eGFR of these patients, as well as on their different stages of chronic kidney disease. Based on the previous notion, we could not differentiate between the cause of renal impairment (chronic kidney disease versus obstructive tumor). Moreover, considering that we assess a cohort study of more than 15 years, we could not evaluate the effect of the evolution of surgical techniques and perioperative medical care on postoperative and long-term outcomes.

## Conclusions

The present high-volume, prospective cohort study from a tertiary referral center indicates that, beside increased age and worse oncological status, impaired renal function, lower preoperative hemoglobin values, higher postoperative thrombocyte values, and perioperative complications are independent risk factors for mortality in the long-term in patients undergoing RC. Therefore, physicians performing RC should be aware that not only the baseline patients’ characteristics but also the occurrence of any perioperative complications can adversely affect long-term survival.

## Data Availability

NP had full access to all the data in the study and takes responsibility for the integrity of the data and the accuracy of the data analysis.
